# Case I.—Passage of Hard Non-Fusible Phosphate of Lime Calculus from Bladder. Case II.—Polyuria Due to Tubercular Peritonitis*Read before the Richmond Academy of Medicine and Surgery, April 23, 1895.

**Published:** 1895-05

**Authors:** William S. Gordon

**Affiliations:** President Richmond Academy of Medicine and Surgery, Professor of Physiology University College of Medicine, Richmond, Va.


### CASE I.—PASSAGE OF HARD NON-FUSIBLE PHOSPHATE OF LIME CALCULUS FROM BLADDER. CASE II.—POLYURIA DUE TO TUBERCULAR PERTONITIS.*

By WILLIAM S. GORDON, Richmond, Va.

President Richmond Academy of Medicine and Surgery, Professor of Physiology University College of Medicine, Richmond, Va.

I wish to report two cases, each presenting one or more noteworthy points.

Case I.—The patient was a young man about twenty-four years of age, who consulted me in November, 1894, for a dull, deep-seated pain in the left lumbar region. His health record up to that time was good. Suspecting, from the nature of his symptoms, that the kidney was the seat of trouble, I made a careful urinary analysis; but finding no pathological condition, prescribed palliative measures and awaited results.

On February 24th last, he was seized with severe left lumbar pain, paroxysmal, characteristic of nephritic colic and accompanied with nausea, vomiting, constipation, slight jaundice, and moderate but irregular rise of temperature.

I was called to him on February 26th, and the diagnosis made of the descent of a calculus from the pelvis of one of the kidneys. The symptoms pointed at first to the left kidney as the one involved, but subsequently the pain extended across the back, and centered mostly in the right side towards the termination of the attack. Cathartics, hot applications, and baths, alkaline diuretics, demulcents and morphine comprised the treatment. The suffering was at times intense, giving rise to epileptoid seizures.

On the morning of March 5th, the calculus started on its urethral journey—having passed into the bladder a day or two previously—and dropped into the vessel after the patient had struggled with it for fifteen minutes. He celebrated the event, much to my regret, by sitting up in bed and whittling upon the stone with a penknife; so that I cannot show it to you in its entirety.

*Read before the Richmond Academy of Medicine and Surgery, April 23, 1895.

My own analysis of the stone proving unsatisfactory, Mr. Hugh Blair kindly undertook the task, and after some difficulty proved it to be an unusually hard, non-fusible phosphate of lime calculus. It is one of the largest I have known to pass from the pelvis of the kidney, weighing eight grains. It must have weighed originally about twelve grains.

Since his attack the patient has suffered from obstinate biliousness, but is now rapidly regaining his health.

Subsequent inquiries reveal that for two years or more he resided in a marked limestone region, drinking the water, and being troubled with more or less indigestion. This is stated for what it is worth. During the attack the urine was excessively acid, scant, and bile colored, but otherwise normal. The patient says that, previous to the attack, he had been passing a brick-dust deposit, and that several days before the passage of the large calculus, a small, though softer one had appeared at the meatus. I had no opportunity of examining the deposit of the smaller calculus and am therefore in doubt as to whether the phosphatic preceded a uric acid condition in this case.

It would be instructive to know what relation, if any, or association there was between the two. The future may solve the problem.

CASE II.—A colored child, six years of age, was brought to me last summer with dyspepsia, debility and some emaciation. The symptoms pointed at that time to gastric disturbance, for which I prescribed, charging the mother to inform me within a reasonable time of the patient's condition. I heard nothing more of the case until December last, when she was brought to me again with symptoms about the same in character, but more pronounced. I prescribed an alterative tonic and requested that the child be brought to me again for closer examination.

This was not done, and I did not see the case again until March 1st of this year. The symptoms were as follows: Anorexia, anæmia, marked emaciation and debility, irregular chills and fever, œdema of the feet and legs, diarrhœa, and extreme thirst and polyuria. The lungs and heart were normal, and no family history of tuberculosis was obtainable. The temperature on March 2d, at 12:30 P. M., was normal, and the pulse one hundred or more.

The mother's statement that the child drank from half a gallon to one gallon of water a day and passed an equal amount of urine, coupled with the fact that I was not permitted to watch the case, led me to suspect diabetes mellitus. The analysis of the urine, however, showed it to be almost like pure water, the specific gravity being 1,000 with an acid reaction. No phosphates were precipitated, but the sulphates and chlorides were noticeable. At my next visit I examined the abdomen, finding it swollen and tender; and the diagnosis of tubercular peritonitis was easily made.

Slight improvement took place under alteratives and tonics, with a decrease of the thirst and polyuria; but treatment soon became inefficacious, and the child died from exhaustion on the 9th of this month.

It is well-known that so-called diabetes insipidus may be caused by certain abdominal diseases. Polyuria is probably a better term to express the renal condition associated with abdominal trouble, inasmuch as true diabetes insipidus is supposed to be dependent upon direct or reflex excitement of certain areas in the bulb. Tubercular disease affecting the bulb in any way would cause polyuria through local dilatation of the renal blood vessels; but just as likely this would be counterbalanced or overcome by the contraction of the renal vessels, which is disproportionate to the contraction of the abdominal vessels, dependent upon the morbid stimulation of the cord. We are thus led to believe that abdominal disease leads to polyuria by pressure upon the abdominal blood vessels, or stimulation of the abdominal vaso-constrictor nerves, both of which conditions would increase the renal blood pressure and thus result in polyuria.

The practical point to bear in mind is that polyuria may be one of the most prominent symptoms of tubercular peritonitis, even when the abdominal symptoms are not marked. The tympanites in this case was not always very noticeable, and the other abdominal symptoms could have been caused by other morbid states. In such cases the underlying morbid condition may escape detection unless great care be exercised.

